# Association of two microRNA polymorphisms miR-27 rs895819 and miR-423 rs6505162 with the risk of cancer

**DOI:** 10.18632/oncotarget.16443

**Published:** 2017-03-22

**Authors:** Hong Zhang, Yafei Zhang, Xixi Zhao, Xingcong Ma, Wanjun Yan, Wen Wang, Zitong Zhao, Qian Yang, Xi Sun, Hui Luan, Xiaoyan Gao, Shuqun Zhang

**Affiliations:** ^1^ Department of Oncology, Second Affiliated Hospital, School of Medicine, Xi’an Jiaotong University, Xi’an, Shaanxi, China; ^2^ Department of General Surgery, Second Affiliated Hospital, School of Medicine, Xi’an Jiaotong University, Xi’an, Shaanxi, China; ^3^ Department of Integrated traditional Chinese and Western Medicine, Second Affiliated Hospital, School of Medicine, Xi’an Jiaotong University, Xi’an, Shaanxi, China; ^4^ Department of Cardiology, First Affiliated Hospital, School of Medicine, Xi’an Jiaotong University, Xi’an, Shaanxi, China

**Keywords:** cancer, rs895819, rs6505162, meta-analysis

## Abstract

Many studies have been conducted to investigate the association between miR-27 rs895819 A > G and miR-423 rs6505162 C > A and cancer risk; however, the results are not consistent. In order to acquire a more precise assessment of the correlation, we performed this meta-analysis. We searched PubMed, EMBASE and Web of Science databases to identify eligible studies. Pooled odds ratios (ORs) and 95% confidence intervals (CIs) were applied to evaluate the correlation of these two microRNA polymorphisms with cancer risk. Forty-five eligible studies from thirty-five articles were included in our analysis. The results showed that rs895819 was associated with a decreased cancer risk in Caucasians (AG vs. AA: OR = 0.87, 95% CI = 0.79-0.96; GG+AG vs. AA: OR = 0.89, 95% CI = 0.81-0.98). When grouped by ethnicity, an increased risk was observed in colorectal cancer (G vs. A: OR = 1.19, 95% CI = 1.08-1.32; GG vs. AA: OR = 1.58, 95% CI = 1.28-1.96; GG vs. AG+AA: OR = 1.58, 95% CI = 1.29-1.93), while a decreased risk was found in breast cancer (G vs. A: OR = 0.93, 95% CI = 0.87-0.99; GG+AG vs. AA: OR = 0.91, 95% CI = 0.83-0.99). For rs6505162, a significantly decreased cancer risk was observed in lung cancer under all five genetic models. To summarize, our results indicated that rs895819 was a protective factor for cancer in Caucasians and could increase colorectal cancer risk but decrease breast cancer risk. Moreover, rs6505162 was a protective factor for lung cancer.

## INTRODUCTION

MicroRNAs (miRNAs) are a class of small, endogenous, non-coding, single-stranded, highly conserved and tissue-specific RNA molecules which take part in the regulation of target mRNAs expression at the post-transcriptional level [1, 2]. By binding to the complementary sequence of the 3’ untranslated region of the specific mRNAs, miRNAs could increase or suppress the expression of multiple target genes, including cancer-associated genes, and thus are involved in many physiological and pathological process, such as cell proliferation, cell differentiation, cell apoptosis, carcinogenesis and development and so on [3, 4].

As the most common type of genetic variation in the human genome, single nucleotide polymorphisms (SNPs) occurring in miRNA genes have been found to be able to affect miRNA expression and function by interfering the interaction between miRNAs and their corresponding target mRNAs. A large number of studies have demonstrated that SNPs in the miRNA genes are associated with the occurrence of various diseases, such as cancer, so microRNA polymorphisms are considered to be a potentially important mechanism in the acquisition of cancer susceptibility [5, 6]. In recent years, some SNPs in miRNAs have been identified as oncogenes of many different types of cancer and found to play an important role in initiation and development of malignancies[7, 8].

However, for two common functional microRNA polymorphisms miR-27 rs895819 and miR-423 rs6505162, although many case-control studies involving various cancer types have been performed among different ethnic populations, there is currently no consensus on whether there exists an association between these two microRNA polymorphisms and cancer risk because of inconsistent results published studies reported. In addition, many relevant case-control studies on this theme have been published recently, which will expand the sample size and may have an impact on the evaluation of the association [9–13]. Therefore, we carried out an updated and systematic meta-analysis including those newly published articles to further assess the correlation of rs895819 and rs6505162 with cancer risk based on all available eligible studies at present.

## RESULTS

### Characteristics of included studies

**Figure 1:**
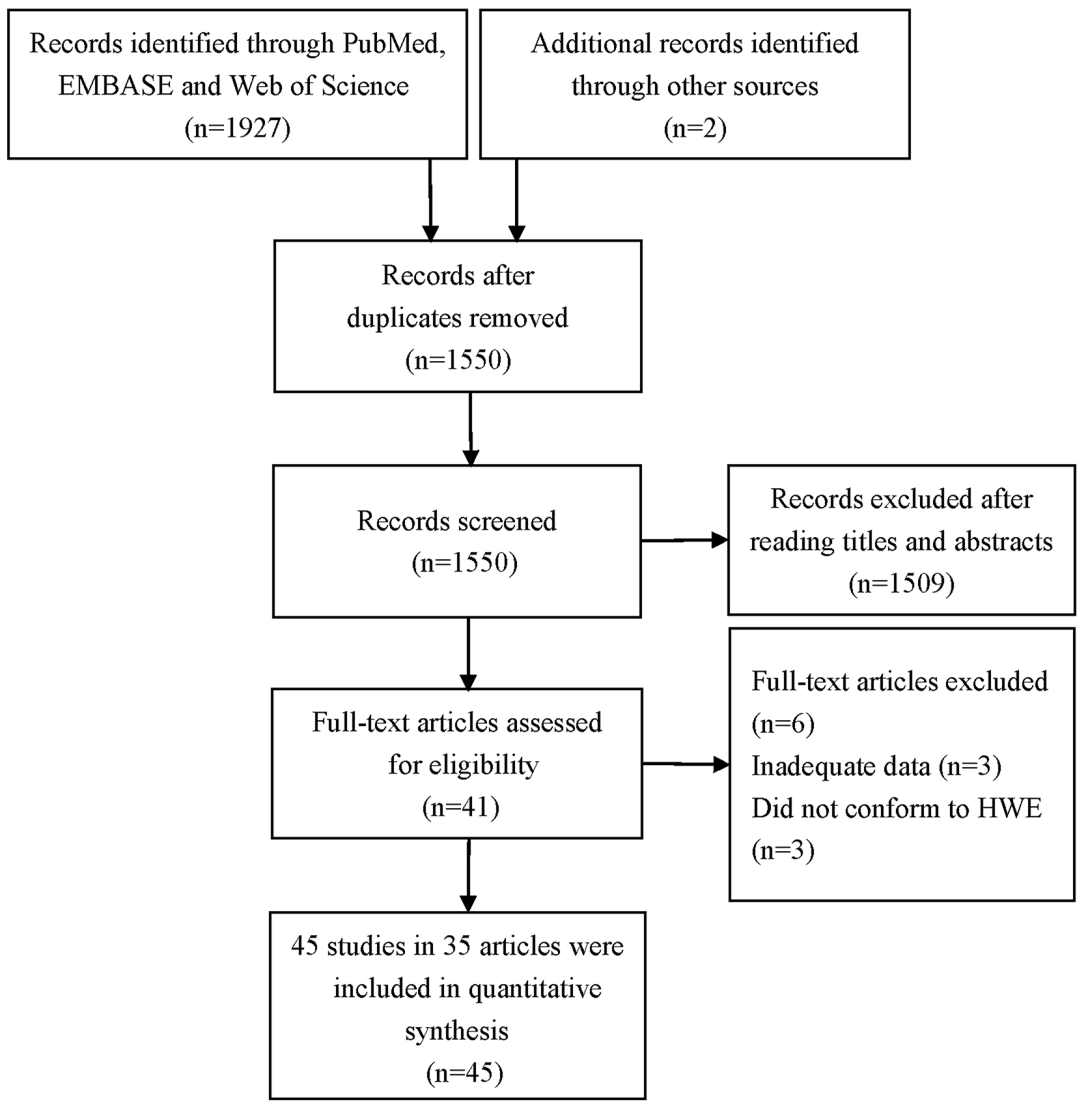
Flow diagram of the selection process of included studies in this meta-analysis

The selection process of eligible studies is presented in Figure 1. A total of 1929 potentially relevant articles were preliminarily identified though a systematic publication search. After excluding duplicate literatures and further carefully reading titles and abstracts of the remaining studies, forty-one articles were performed full-text review for eligibility, among which six articles were excluded for the following reasons: three articles did not have sufficient data; three articles did not conform to HWE. Thus thirty-five articles were considered to meet our inclusion criteria [9–43]. Among them, seven articles involved not only rs895819 but also rs6505162 [9, 11, 14–15, 19, 29, 36]. Moreover, the study by Kontorovich et al. [36] was performed in both breast cancer and ovarian cancer, so we treated the study as two independent investigations. Ultimately, forty-five studies from thirty-five articles were included in quantitative synthesis. The main characteristics of the included studies are listed in Table 1. Of all the included studies, fifteen focused on breast cancer, eight on esophageal squamous cell carcinoma, six on gastric cancer, five on colorectal cancer, four on lung cancer, two on ovarian cancer, and one each on prostate cancer, gallbladder cancer, cervical cancer, renal cell cancer and hepatocellular carcinoma. In addition, thirty-five studies were carried out among Asians, and eight among Caucasians. A variety of genotyping methods including PCR-RFLP, MassARRAY, Taqman, Sequencing, PCR-LDR, SNaPshot, Allele-specific PCR, ARMS-PCR and HRM were applied in the included studies of this meta-analysis. Moreover, twenty studies were considered as moderate-quality studies (Newcastle-Ottawa Scale scores of these studies were 4-6), and other twenty-five studies were considered as high-quality studies (Newcastle-Ottawa Scale scores of these studies were 7 or above). Genotype distributions of all forty-five studies in the controls were in agreement with Hardy-Weinberg equilibrium (HWE).

**Table 1:** The main characteristics of included studies in the meta-analysis

First author	Year	Country	Ethnicity	Cancer type	Genotyping methods	Source of controls	Case (n)			Control (n)			HWE	NOS
							AA	AB	BB	AA	AB	BB		
rs895819														
Jiang	2016	China	Asian	GC	MassARRAY	HB	480	356	59	537	389	62	0.447	7
Jiang	2016	China	Asian	CRC	TaqMan	HB	245	176	87	275	222	65	0.053	7
Yin	2016	China	Asian	LC	TaqMan	HB	321	217	37	318	252	38	0.199	7
Bian	2015	China	Asian	CRC	TaqMan	HB	199	143	70	205	166	41	0.389	7
He	2015	China	Asian	BC	MassARRAY	PB	251	165	34	232	181	37	0.839	7
Gupta	2015	India	Asian	GBC	TaqMan	PB	75	81	20	26	24	3	0.399	6
Nikolic	2015	Serbia	Caucasian	PC	ASPCR	PB	151	172	30	152	137	19	0.101	6
Qi	2015	China	Asian	BC	TaqMan	PB	101	159	61	95	139	56	0.686	7
Zhang	2015	China	Asian	BC	MassARRAY	PB	196	150	30	106	70	14	0.605	7
Yin	2015	China	Asian	LC	TaqMan	HB	138	103	17	167	125	18	0.391	6
Cao	2014	China	Asian	CRC	PCR-RFLP	PB	92	113	49	114	93	31	0.089	7
Kupcinskas	2014	Countries^a^	Caucasian	CRC	TaqMan	HB	87	79	25	203	191	34	0.235	4
Kupcinskas	2014	Countries^b^	Caucasian	GC	TaqMan	HB	181	144	38	156	164	30	0.151	6
Song	2014	China	Asian	GC	Sequencing	HB	105	124	49	131	111	36	0.110	7
Xiong	2014	China	Asian	CC	PCR-LDR	HB	48	40	15	223	170	24	0.255	7
Zhang	2014	China	Asian	ESCC	SNaPshot	PB	613	414	82	719	466	90	0.226	7
Ma	2013	China	Asian	BC	MassARRAY	HB	97	76	16	106	70	14	0.605	6
Wei	2013	China	Asian	ESCC	MassARRAY	HB	216	143	20	208	139	30	0.322	6
Zhang	2013	China	Asian	BC	Sequencing	PB	152	96	16	137	103	15	0.447	7
Catucci	2012	Italy	Caucasian	BC	TaqMan	HB	547	388	90	803	633	157	0.051	5
Hezova	2012	Czech	Caucasian	CRC	TaqMan	HB	88	86	23	93	94	25	0.867	6
Shi	2012	China	Asian	RCC	TaqMan	HB	334	213	47	288	262	50	0.373	7
Zhang	2012	China	Asian	BC	PCR-RFLP	PB	60	144	41	75	109	59	0.123	7
Zhou	2012	China	Asian	GC	MassARRAY	HB	166	122	7	214	167	32	0.941	5
Sun	2010	China	Asian	GC	PCR-RFLP	HB	115	135	54	145	119	40	0.053	6
Kontorovich	2010	Israel	Asian	BC	MassARRAY	HB	98	78	11	101	82	15	0.769	5
Kontorovich	2010	Israel	Asian	OC	MassARRAY	HB	43	34	3	101	82	15	0.769	5
Yang	2010	Germany	Caucasian	BC	Sequencing	PB	576	486	127	605	660	151	0.142	7
rs6505162														
Yin	2016	China	Asian	LC	TaqMan	HB	389	166	20	368	205	35	0.366	7
Morales	2016	Chile	Caucasian	BC	TaqMan	HB	125	229	86	284	385	138	0.700	5
Jiang	2016	China	Asian	GC	MassARRAY	HB	593	255	32	656	288	41	0.192	7
Shen	2016	China	Asian	ESCC	SNaPshot	PB	920	421	59	1421	680	84	0.814	7
Zhang	2015	China	Asian	BC	MassARRAY	PB	231	131	20	110	69	10	0.847	7
Zhu	2015	China	Asian	ESCC	MassARRAY	PB	99	122	21	109	140	31	0.159	7
Yin	2015	China	Asian	LC	TaqMan	HB	177	74	7	190	106	14	0.872	6
He	2015	China	Asian	BC	MassARRAY	PB	292	142	16	299	129	22	0.103	7
Ma	2014	China	Asian	HCC	MassARRAY	HB	652	297	42	643	313	30	0.273	7
Ma	2013	China	Asian	BC	MassARRAY	HB	127	57	8	110	69	10	0.847	6
Umar	2013	India	Asian	ESCC	ARMS-PCR	HB	90	132	67	96	143	70	0.233	7
Yin	2013	China	Asian	ESCC	PCR-LDR	HB	374	197	29	425	207	19	0.299	7
Wang	2013	Countries^c^	Black	ESCC	TaqMan	PB	207	128	16	376	184	12	0.052	6
Wang	2013	Countries^c^	Mixed	ESCC	TaqMan	PB	89	84	14	198	188	34	0.249	5
Smith	2012	Australia	Caucasian	BC	HRM	HB	60	95	24	52	80	42	0.307	7
Kontorovich	2010	Israel	Caucasian	BC	iPLEX	PB	68	88	34	55	102	49	0.899	5
Kontorovich	2010	Israel	Caucasian	OC	iPLEX	PB	31	26	22	55	102	49	0.899	5

A: the major allele; B: the minor allele; ASPCR: allele-specific PCR; GC: gastric cancer; CRC: colorectal cancer; LC: lung cancer; BC: breast cancer; GBC: gallbladder cancer; PC: prostate cancer; CC: cervical cancer; ESCC: esophageal squamous cell carcinoma; RCC: renal cell cancer; OC: ovarian cancer; HCC: hepatocellular carcinoma; PB: population based; HB: hospital based; a: Lithuania and Latvia; b: German, Lithuanian and Latvian; c: South Africa.

### Meta-analysis results

**Figure 2:**
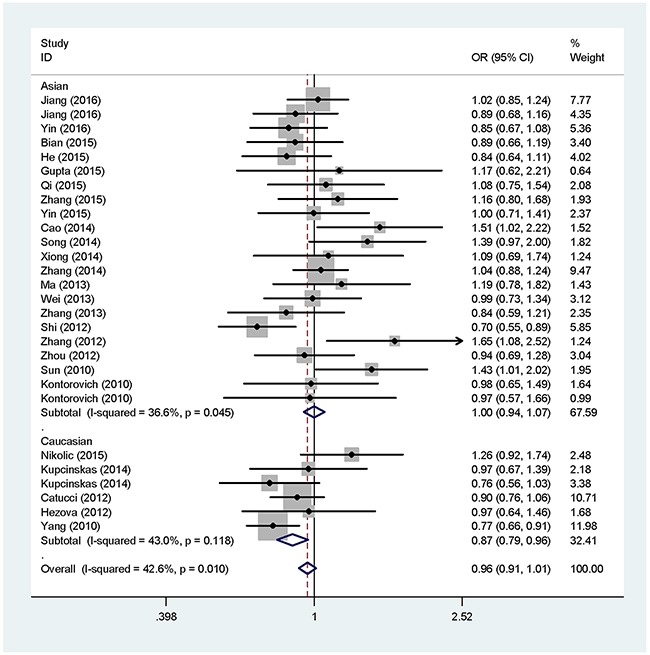
Forest plot of the association between rs895819 and cancer risk in subgroup analysis by ethnicity under heterozygote model

**Figure 3:**
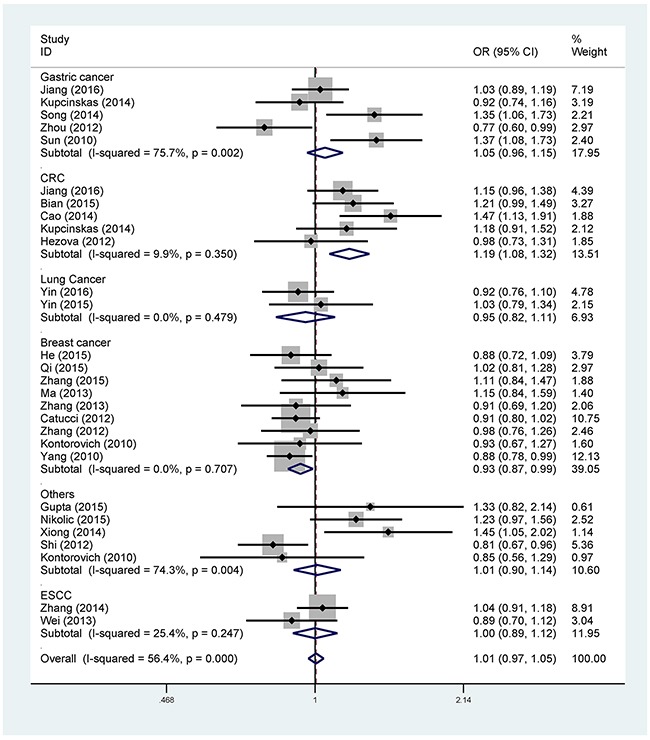
Forest plot of the association between rs895819 and cancer risk in subgroup analysis by cancer type under allele contrast model

The main meta-analysis results of the correlation between rs895819 A > G and cancer susceptibility are listed in Table 2. In the overall analysis, we couldn't detect an association of the rs895819 polymorphism with cancer risk in all five genetic models. However, when analysis was stratified by ethnicity, a decreased cancer risk was observed in the Caucasian population (AG vs. AA: OR = 0.87, 95% CI = 0.79-0.96; GG+AG vs. AA: OR = 0.89, 95% CI = 0.81-0.98), while no significant correlation was found among Asians in all the genetic models (Table 2) (Figure 2). Moreover, the stratified analysis by cancer type revealed that rs895819 was associated with an increased risk of colorectal cancer (G vs. A: OR = 1.19, 95% CI = 1.08-1.32; GG vs. AA: OR = 1.58, 95% CI = 1.28-1.96; GG vs. AG+AA: OR = 1.58, 95% CI = 1.29-1.93), and a decreased risk of breast cancer (G vs. A: OR = 0.93, 95% CI = 0.87-0.99; GG+AG vs. AA: OR = 0.91, 95% CI = 0.83-0.99) (Table 2) (Figure 3). Nevertheless, we didn't find an association of the rs895819 polymorphism with risks of lung cancer, gastric cancer, prostate cancer, gallbladder cancer, cervical cancer, renal cell cancer, ovarian cancer and esophageal squamous cell carcinoma. We also performed the subgroup analysis by source of controls and the result indicated that there was no association between rs895819 and cancer risk in either population-based subgroup or hospital-based subgroup.

**Table 2:** Meta-analysis results of the association between rs895819 and cancer risk

Variables	N^a^	G vs. A		GG vs. AA		AG vs. AA		GG+AG vs. AA		GG vs.AG+AA	
		OR (95% CI)	I^2^ (%)	OR (95% CI)	I^2^ (%)	OR (95% CI)	I^2^(%)	OR (95% CI)	I^2^ (%)	OR (95% CI)	I^2^(%)
Overall	28	1.03 (0.97,1.10)^b^	56.4	1.12 (0.97,1.29)^b^	52.2	0.99 (0.91,1.06)^b^	42.6	1.01 (0.94,1.09)^b^	49.7	1.11 (0.97,1.26)^b^	49.6
Ethnicity											
Asians	22	1.05 (0.97,1.13)^b^	55.9	1.13 (0.95,1.34)^b^	54.2	1.02 (0.93,1.11)^b^	36.6	1.04 (0.95,1.13)^b^	44.9	1.10 (0.93,1.30)^b^	54.9
Caucasians	6	0.94 (0.88,1.01)	48.7	0.98 (0.83,1.15)	36.8	0.87 (0.79,0.96)	43.0	0.89 (0.81,0.98)	49.0	1.05 (0.90,1.22)	23.1
Cancer type											
BC	9	0.93 (0.87,0.99)	0.0	0.90 (0.78,1.04)	0.0	0.97 (0.84,1.11)^b^	50.3	0.91 (0.83,0.99)	33.7	0.92 (0.80,1.05)	0.0
CRC	5	1.19 (1.08,1.32)	9.9	1.58 (1.28,1.96)	0.0	0.98 (0.85,1.14)	29.3	1.11 (0.97,1.27)	23.0	1.58 (1.29,1.93)	0.0
LC	2	0.95 (0.82,1.11)	0.0	1.02 (0.69,1.51)	0.0	0.90 (0.74,1.09)	0.0	0.91 (0.76,1.10)	0.0	1.07 (0.73,1.57)	0.0
GC	5	1.06 (0.87,1.29)^b^	75.7	1.08 (0.68,1.70)^b^	74.7	1.06 (0.86,1.31)^b^	60.8	1.08 (0.85,1.36)^b^	70.9	1.05 (0.71,1.56)^b^	68.6
ESCC	2	1.00 (0.89,1.12)	25.4	0.95 (0.72,1.26)	54.1	1.03 (0.89,1.19)	0.0	1.02 (0.88,1.17)	0.0	0.94 (0.72,1.24)	52.4
Others	5	1.09 (0.84,1.41)^b^	74.3	1.35 (0.73,2.49)^b^	69.0	0.99 (0.75,1.31)^b^	59.5	1.05 (0.77,1.43)^b^	69.4	1.35 (0.80,2.28)^b^	60.5
Source of controls											
PB	10	1.04 (0.94,1.15)^b^	55.9	1.05 (0.91,1.21)	22.2	1.06 (0.90,1.24)^b^	63.7	1.07 (0.92,1.24)^b^	63.8	1.03 (0.90,1.18)	15.2
HB	18	1.03 (0.94,1.11)^b^	59.0	1.12 (0.91,1.37)^b^	61.7	0.94 (0.88,1.01)	21.4	0.99 (0.90,1.08)^b^	40.5	1.14 (0.94,1.37)^b^	58.9

a: number of studies; b: calculating based on random-effects model; BC: breast cancer; CRC: colorectal cancer; LC: lung cancer; GC: gastric cancer; ESCC: esophageal squamous cell carcinoma.

**Figure 4:**
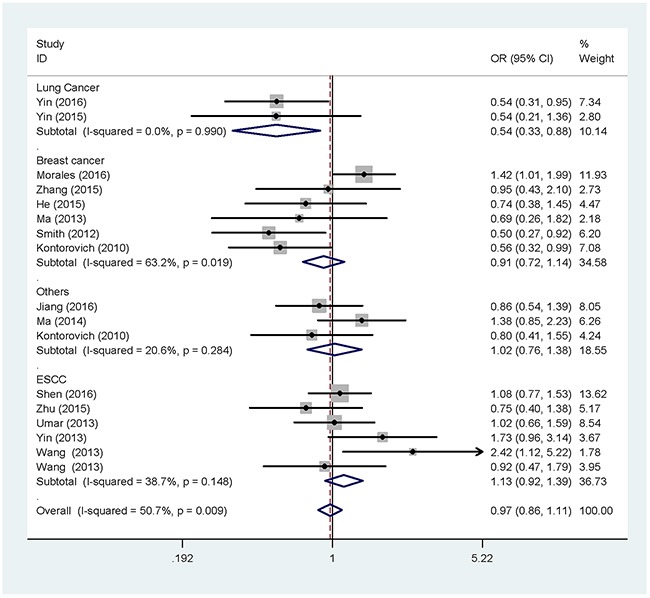
Forest plot of the association between rs6505162 and cancer risk in subgroup analysis by cancer type under homozygote model

For rs6505162 C > A, no significant risk association was observed in the overall analysis. Similarly, when grouped by ethnicity, we couldn't detect a correlation of the rs6505162 polymorphism with cancer risk in either Caucasians or Asians. However, after subgroup analysis by cancer type, the rs6505162 polymorphism was found to be associated with a decreased risk of lung cancer in all five genetic models (A vs. C: OR = 0.75, 95% CI = 0.63-0.88; AA vs. CC: OR = 0.54, 95% CI = 0.33-0.88; AC vs. CC: OR = 0.76, 95% CI = 0.62-0.93; AA+AC vs. CC: OR = 0.73, 95% CI = 0.60-0.89; AA vs. AC+CC: OR = 0.59, 95% CI = 0.37-0.95) (Table 3) (Figure 4). Besides, subgroup analysis by source of controls revealed that there was no correlation between rs6505162 and cancer susceptibility in either population-based subgroup or hospital-based subgroup.

**Table 3:** Meta-analysis results of the association between rs6505162 and cancer risk

Variables	N^a^	A vs. C		AA vs. CC		AC vs. CC		AA+AC vs. CC		AA vs. AC+CC	
		OR (95% CI)	I^2^(%)	OR (95% CI)	I^2^(%)	OR (95% CI)	I^2^(%)	OR (95% CI)	I^2^(%)	OR (95% CI)	I^2^(%)
Overall	17	0.96 (0.88,1.04)^b^	56.1	0.93 (0.77,1.14)^b^	50.7	0.96 (0.87,1.05)^b^	40.2	0.95 (0.86,1.05)^b^	50.6	0.96 (0.81,1.14)^b^	40.6
Ethnicity											
Asians	11	0.96 (0.90,1.02)	35.3	0.96 (0.82,1.13)	27.7	0.94 (0.87,1.01)	0.0	0.94 (0.88,1.01)	16.9	0.98 (0.84,1.15)	19.2
Caucasians	4	0.88 (0.66,1.17)^b^	78.3	0.7 7(0.44,1.35)^b^	77.0	0.85 (0.54,1.36)^b^	78.0	0.84 (0.54,1.30)^b^	79.3	0.86 (0.56,1.31)^b^	69.7
Cancer type											
LC	2	0.75 (0.63,0.88)	0.0	0.54 (0.33,0.88)	0.0	0.76 (0.62,0.93)	0.0	0.73 (0.60,0.89)	0.0	0.59 (0.37,0.95)	0.0
BC	6	0.91 (0.75,1.09)^b^	67.3	0.79 (0.52,1.19)^b^	63.2	1.03 (0.90,1.19)	51.7	0.93 (0.74,1.18)^b^	62.5	0.87 (0.71,1.07)^b^	47.1
ESCC	6	1.04 (0.96,1.13)	33.9	1.13 (0.92,1.39)	38.7	1.02 (0.92,1.13)	0.0	1.04 (0.94,1.14)	5.6	1.12 (0.92,1.37)	30.8
Others	3	0.98 (0.88,1.09)	0.0	1.02 (0.76,1.38)	20.6	0.92 (0.81,1.06)	63.7	0.94 (0.83,1.07)	43.9	1.14 (0.85,1.52)	5.3
Source of controls											
PB	8	0.98 (0.91,1.06)	40.0	0.93 (0.76,1.13)	36.2	0.97 (0.88,1.08)	44.1	0.98 (0.89,1.08)	45.9	0.98 (0.81,1.19)	22.0
HB	9	0.94 (0.83,1.06)^b^	67.7	0.93 (0.69,1.26)^b^	61.9	0.96 (0.88,1.05)	43.7	0.94 (0.82,1.09)^b^	58.7	0.9 3(0.72,1.21)^b^	55.5

a: number of studies; b: calculating based on random-effects model; LC: lung cancer; BC: breast cancer; ESCC: esophageal squamous cell carcinoma.

### Sensitivity analysis

In order to evaluate the stability of our results, we performed the sensitivity analysis to assess the effect of each individual study on the pooled ORs. After sequentially excluding each eligible studies, the corresponding ORs were not substantially changed, showing that the results of this meta-analysis are statistically stable.

### Heterogeneity analysis

Q test and I^2^ test were used to assess the heterogeneity among studies. When obvious between-study heterogeneity (*P* value of Q test was less than 0.1 or I^2^ value was greater than 75%) was observed in the overall pooled analysis or subgroup analysis, the random-effects model would be applied because it could generate wider confidence intervals. Otherwise, we would select fixed-effects model to conduct related data analysis.

### Publication bias

**Figure 5:**
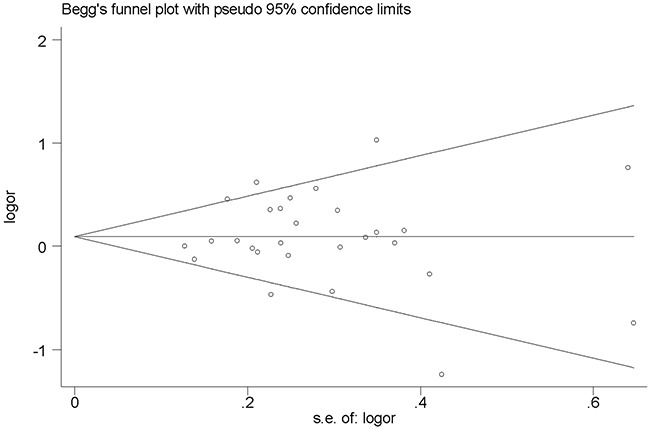
Begg's funnel plot of rs895819 in recessive model

The Begg's funnel plot and Egger's test were performed to examine the publication bias in this meta-analysis. For rs895819, no publication bias was found in allele contrast model, homozygote model and recessive model, and the shape of the funnel plots appeared to relatively symmetrical (Figure 5). However, there was evidence of significant publication bias in the other two genetic models (*P* values of Begg's test and Egger's test were less than 0.05). Similarly, we could not observe a publication bias for the rs6505162 polymorphism in three of five comparison models including homozygote model, heterozygote model and recessive model. Interestingly, for both allele contrast model and recessive model, *P* values of Egger's test in these two genetic models were all > 0.05, while that of Begg's test were all < 0.05, which might be due to small sample size and limited number of studies included in this meta-analysis. Therefore, there might exist publication bias in our study.

## DISCUSSION

In the current meta-analysis, a total of forty-five eligible case-control studies from thirty-five articles were included and their pooled results were used to assess the association of microRNA polymorphisms including miR-27 rs895819 and miR-423 rs6505162 with cancer susceptibility. We found that the rs895819 polymorphism was associated with a decreased cancer risk in Caucasians and breast cancer, while a significantly increased risk for developing cancer could be detected in colorectal cancer under allele contrast model, homozygote model and recessive model. In addition, for the rs6505162 polymorphism, a significantly decreased risk for cancer could be observed in lung cancer when data were stratified by cancer type.

As an important member of the microRNAs family, miR-27 is located in the intergenic region of chromosome 19 and is involved in the occurrence, invasion and metastasis of many types of cancer by acting as an oncogene. The miR-27 displays its oncogenic property through regulating the expression of target genes including FOXO1 [44], BTG2 [45], PHB [46] and other cancer-related genes [47]. Studies have reported that genetic mutations in miRNA precursors (pre-miRNA) can alter miRNA expression levels and their binding with several nuclear factors during miRNA processing [48]. Consequently, rs895819, an A to G nucleotide substitution occurring in pre-miR-27a, can down-regulate miR-27 by influencing its expression and/or maturation, and thus may lead to a reduction of individual susceptibility to cancer. Our results support this inference. In our study, we found that rs895819 could decrease the risk of breast cancer in allele contrast model and dominant model.

In addition to being able to function as an oncogene, many studies have demonstrated that miR-27a can also play an essential role as a tumor-suppressor gene in the development and progression of various cancer. For example, Bao and colleagues found that miR-27a could act as a tumor suppressor for colorectal cancer through inhibiting colon cancer cell proliferation and migration, and inducing colon cancer cell apoptosis [49]. As described in the previous paragraph, the rs895819 polymorphism can down-regulate the expression of miR-27, which will affect the tumor suppressor function of miR-27, so rs895819 may be able to promote the occurrence and development of colorectal cancer by attenuating miR-27's tumor suppressive effect. Therefore, rs895819 may be a risk factor for colorectal cancer, which is consistent with the results of our study. In our meta-analysis, the rs895819 polymorphism was found to be associated with an increased risk of colorectal cancer in allele contrast model, homozygote model and recessive model. In addition, three latest publications reported similar results [50–52], which will make our conclusions more credible. Moreover, when analysis was stratified by ethnicity, a decreased cancer risk could be detected among Caucasians, indicating that the distinction in genetic backgrounds among different ethnic groups might have an impact on cancer susceptibility, which was in accordance with the results of five previously published studies [52–56] and further demonstrated the reliability of our results.

For rs6505162, our results showed that there was no association between rs6505162 and cancer risk in the overall pooled analysis. When grouped by race, similar results could still be observed among both Asian populations and Caucasian populations. However, in the subgroup analysis by cancer type, a significantly decreased cancer risk was found in lung cancer, demonstrating that rs6505162 polymorphism might be a protective factor for lung cancer. But considering that only two studies were included in the analysis, we should treat the result with caution. Our results were different from a previously published study by Hu et al. [57], which included eight studies and found that rs6505162 was a risk factor for cancer, especially for bladder cancer. This discrepancy may be due to differences in sample sizes and ethnic groups: compared with his study, our study included seventeen eligible studies, which would expand the sample size more than twice and thus get a more credible assessment of the association.

However, some limitations of this meta-analysis should be addressed. Firstly, several important individual information such as age, sex, environmental factor was not available for most of studies included in our meta-analysis, so we couldn't carry out a more detailed analysis grouped by other non-genetic risk factors of cancer. Secondly, there existed a moderate degree of heterogeneity among the included studies, however, when analysis stratified by cancer type or race, between-study heterogeneity obviously reduced, demonstrating that heterogeneity might partly stem from differences in cancer types and ethnicities. Thirdly, many kinds of genotyping methods were applied in the included studies of our meta-analysis, which might have a potential impact on the results. Fourthly, publication bias existed for both rs895819 and rs6505162 because only studies collected into PubMed, EMBASE and Web of Science databases and published in English were selected into our meta-analysis. Those literatures not collected into these three databases or published not in English were not included in our analysis, which might contain other eligible studies and thus cause publication bias. Therefore, in view of these limitations, we should cautiously treat the results of our study.

In conclusion, our results indicate that miR-27 rs895819 is a protective factor for developing cancer in Caucasian populations and could increase colorectal cancer risk but decrease breast cancer risk. For miR-423 rs6505162, it could decrease the risk of lung cancer. More multi-center large-scale case-control studies with larger sample sizes are needed to confirm our results.

## MATERIALS AND METHODS

### Literature and search strategy

A comprehensive literature search of all eligible studies published before October 21, 2016 was conducted in PubMed, EMBASE and Web of Science electronic databases. During our searching process, the following retrieval items were applied: (“microRNA 27” OR “microRNA-27” OR “miR-27” OR “rs895819” OR “microRNA 423” OR “microRNA-423” OR “miR-423” OR “rs6505162”) AND (“polymorphism” OR “SNP” OR “variation” OR “locus” OR “mutation”) AND (“cancer” OR “tumor” OR “malignancy” OR “carcinoma” OR “neoplasm”).

### Criterion for study selection

All eligible studies included in this meta-analysis must meet the following criteria: (1) evaluating the association of rs895819/rs6505162 with cancer risk; (2) case-control design; (3) providing sufficient genotyping data for estimating odds ratios (ORs) and 95% confidence intervals (CIs); (4) genotype frequencies of subjects in controls were in accordance with Hard-Weinberg equilibrium (HWE); (5) all cancer cases were confirmed by pathology. The exclusion criteria were as follows: (1) not case-control studies; (2) case reports, comments or review articles; (3) duplicate articles; (4) the control groups did not conform to HWE; (5) lack of enough genotyping data.

### Data extraction and quality assessment

Two independent investigators carefully collected the following information from each study included in this meta-analysis: first author, year, country, ethnicity, cancer type, genotyping method, source of controls, number of cases and controls, genotype distribution of cases and controls, and *P* value of Hardy-Weinberg equilibrium (HWE) in controls. Any inconsistency was resolved through discussion until a consensus was reached. Moreover, we also evaluated the methodological quality of included studies based on Newcastle-Ottawa Scale (NOS), which scored studies according to three aspects: selection, comparability, and exposure. Therefore, all studies could be divided into three categories: “low quality” studies (score 0-3); “moderate quality” studies (score 4-6); “high quality” studies (score 7-9).

### Statistical method

The association of rs895819 and rs6505162 with cancer susceptibility was estimated by pooled odds ratios (ORs) and 95% confidence intervals (CIs) under five different genetic models including allele contrast model, homozygote model, heterozygote model, dominant model and recessive model. Both Q-statistical test and I^2^ test were applied to assess the between-study heterogeneity in this meta-analysis. If there was significant heterogeneity among included studies (*P* value of Q statistic was < 0.1 or I^2^ value was > 75%), ORs with corresponding 95% CIs would be calculated using the random-effects model; otherwise, the fixed-effects model would be selected. The subgroup analysis was undertaken according to ethnicity, cancer type (investigations consisting of only one study were merged into the “others” group) and source of controls. We conducted sensitivity analysis by sequentially omitting a single study each time to assess the influence of individual study on the pooled ORs. Moreover, we employed Begg's funnel plot and Egger's test (*P* < 0.05 was considered significant) to assess whether there existed publication bias. Besides, chi-square test was applied to calculate *P* value of Hardy-Weinberg equilibrium (HWE) in controls. All data analyses performed in the present analysis were carried out using STATA 12.0 software (StataCorp LP, College Station, TX, USA). All statistical tests were two sided, and *P* < 0.05 indicated statistical significance.
